# A *CsMYB6-CsTRY* module regulates fruit trichome initiation in cucumber

**DOI:** 10.1093/jxb/ery047

**Published:** 2018-02-08

**Authors:** Sen Yang, Yanling Cai, Xingwang Liu, Mingming Dong, Yaqi Zhang, Shuying Chen, Wenbo Zhang, Yujing Li, Min Tang, Xuling Zhai, Yiqun Weng, Huazhong Ren

**Affiliations:** 1 Beijing Key Laboratory of Growth and Developmental Regulation for Protected Vegetable Crops, College of Horticulture, China Agricultural University, Beijing, China; 2 USDA-ARS, Vegetable Crops Research Unit, Horticultural Department, University of Wisconsin-Madison, WI, USA

**Keywords:** *CsMYB6*, *CsTRY*, cucumber, fruit spine, regulatory module, trichome initiation

## Abstract

Fruit epidermal features such as the number and size of trichomes or spines are important fruit quality traits in cucumber production. Little is known about the molecular mechanisms underlying fruit spine formation in cucumber. Here, we report functional characterization of the cucumber *CsMYB6* gene, which encodes a MIXTA-like MYB transcription factor that plays an important role in regulating fruit trichome development. Spatial-temporal expression analyses revealed high-level expression of *CsMYB6* in the epidermis of cucumber ovaries during fruit spine initiation, which was similar to the expression of *CsTRY*, a homolog of the Arabidopsis *TRY* gene that also plays a key role in trichome development. Overexpression of *CsMYB6* and *CsTRY* in cucumber and Arabidopsis revealed that *CsMYB6* and *CsTRY* act as negative regulators of trichome initiation in both species, and that *CsMYB6* acted upstream of *CsTRY* in this process. CsMYB6 was found to bind to the three MYB binding sites inside the promoter region of *CsTRY*, and protein–protein interaction assays suggested that CsTRY also directly interacted with CsMYB6 protein. The results also revealed conserved and divergent roles of CsMYB6 and its Arabidopsis homolog AtMYB106 in trichome development. Collectively, our results reveal a novel mechanism in which the *CsMYB6-CsTRY* complex negatively regulates fruit trichome formation in cucumber.

## Teaser

*CsMYB6* interacts with *CsTRY* and they work cooperatively to suppress fruit trichome initiation in cucumber.

## Introduction

Cucumber (*Cucumis sativus* L., 2*n*=2*x*=14) is one of the most economically important vegetable crops in the world, and it is the main vegetable grown in protected environments in China. The cucumber fruit, a pepo that develops from the ovary and receptacle, is covered with a thick cuticle, tubercules, and trichomes (or spines) (Roth, 1977; Wang *et al.*, 2015). The trichomes are widely distributed on leaves, stems, flowers, tendrils, and ovaries (Chen *et al.*, 2014). Two types of trichomes have been observed on cucumber fruits, both of which are multicellular (Chen *et al.*, 2014; Li *et al.*, 2015). Type I, or bloom trichomes, are small and glandular, and produce fine white powdery secretions that are composed primarily of silicon dioxide (SiO_2_). Type I trichomes give cucumber fruits a coarse outer appearance (Yamamoto *et al.*, 1989; Samuels *et al.*, 1993; Chen *et al.*, 2014; Li *et al.*, 2015). Type II, or spines, are much larger than bloom trichomes. Trichomes can serve as barriers to protect plants from stresses, excessive transpiration, ultraviolet light, herbivores, and parasites (Neal *et al.*, 1989; Bodnaryk, 1996; Mauricio and Rausher, 1997; Eisner *et al.*, 1998; Werker, 2000). Warty fruits have both spines and tubercules on their surface. The presence or absence, number, and size of spines and tubercles are important fruit quality traits that define different cucumber market classes. For example, fruits of North American pickles usually have large but sparse spines. The North China fresh market cucumber, with dense and small spines, is more popular in China fresh markets, while the fruits of the European greenhouse-type or the Mediterranean-type (mini) cucumbers are in general smooth and glossy, and lack visible spines and tubercules (Zhang *et al.*, 2010; Yang *et al.*, 2014; Li *et al.*, 2015; Pan *et al.*, 2015). As such, there is considerable interest in understanding the regulatory mechanisms of fruit spine organogenesis in cucumber in order to enhance breeding programs and the economic value of cucumber production.

The model plant *Arabidopsis thaliana* (Arabidopsis) does not bear warty fruits, and the trichomes on its rosette leaves are unicellular, highly branched, and non-glandular. The molecular mechanisms of Arabidopsis trichome formation have been extensively studied. More than 30 genes have been identified that contribute to different aspects of trichome development (Hülskamp *et al*., 1999; Hülskamp *et al.*, 2004; Pattanaik *et al.*, 2014). Among them, a trimeric complex that is composed of GLABRA1 (GL1; Oppenheimer *et al.*, 1991), TRANSPARENT TESTA GLABRA1 (TTG1; Galway *et al.*, 1994; Walker *et al.*, 1999), and GLABRA 3/ENHANCER OF GLABRA3 (GL3/EGL3; Payne *et al.*, 2000; Szymanski *et al.*, 2000; Zhang *et al.*, 2003) activates trichome initiation by promoting the expression of the genes *GL2* and *EGL2* (Larkin *et al.*, 2003). In this complex, R3MYB transcription factors also play vital roles in controlling trichome formation and development. One of these, TRY, is important because it can compete with GL1 for interaction with GL3/EGL3 and can suppress the initiation of trichome formation (Schellmann *et al.*, 2002; Esch *et al.*, 2003; Zhang *et al.*, 2003).

Compared with Arabidopsis, little is known about the molecular mechanisms of trichome formation in cucumber. So far, only two genes, *Cucumber glabrous-1* (*CsGL1*) [syn. *tiny branched hair* (*tbh*), *micro-trichome* (*Mict*)] and *CsGL3* [syn. *trichome-less* (*Tril*)] (Chen *et al.*, 2014; Li *et al.*, 2015; Pan *et al.*, 2015; Zhao *et al.*, 2015) have been identified. The *csgl1* mutant has no visible trichomes on any above-ground organs, but instead has many extremely small trichomes with a stunted morphology that can be seen only under a scanning electron microscope. *CsGL1* may be involved in cucumber trichome development but not in their initiation (Chen *et al.*, 2014; Li *et al.*, 2015; Pan *et al.*, 2015; Zhao *et al.*, 2015; Liu *et al.*, 2016). The *csgl3* mutant is completely glabrous on all aerial organs, and *CsGL3* may be involved in cucumber trichome initiation (Pan *et al.*, 2015; Wang *et al.*, 2016). More recently, we showed that the *CsTTG1* gene, which encodes a WD-repeat protein, plays an important role in regulating fruit bloom trichome and wart formation in cucumber (Chen *et al.*, 2016). Nonetheless, the regulatory network underlying the formation of cucumber fruit trichomes remains poorly understood.

The *MIXTA* gene in snapdragon (*Antirrhinum majus*) (Noda *et al.* 1994) and its homologs in Arabidopsis, cotton (*Gossypium hirsutum*), and many other species have been reported to be both negative and positive regulators of trichome differentiation (Glover *et al.*, 1998; Perez-Rodriguez *et al.*, 2005; Baumann *et al.*, 2007; Jaffé *et al.*, 2007; Jakoby *et al.*, 2008; Machado *et al.*, 2009; Dubos *et al.*, 2010; Walford *et al.*, 2011; Du *et al.*, 2012). *MIXTA-like* genes therefore seem to be involved in trichome development across major core eudicot lineages (Scoville *et al.*, 2011). There are two *MIXTA-like* homologs in the cucumber genome. Phylogenetic analysis of the R2R3MYB family members in cucumber, Arabidopsis, snapdragon, and cotton showed that the MIXTA-like homologs CsMYB6 and CsMYB26 were in the same clade with the Arabidopsis MIXTA-like proteins AtMYB106 (NOK) and AtMYB16 (MIXTA), which have been shown to regulate epidermal cell differentiation, cuticular wax biosynthesis, and trichome morphogenesis (Jakoby *et al.*, 2008; Matus *et al.*, 2008; Wilkins *et al.*, 2009; Li *et al.*, 2012; Yoshimi *et al.*, 2013). In previous transcriptome profiling studies, *CsMYB6* was the only MIXTA-like homolog that was significantly down- regulated in both *csgl1* and *csgl3* mutants (Chen *et al.*, 2014; Li *et al.*, 2015; Zhao *et al.*, 2015). Thus, we hypothesize that *CsMYB6* may be involved in cucumber trichome formation. We developed *CsMYB6*-overexpressing cucumber transgenic lines. We found that CsMYB6 regulates trichome formation by directly interacting with CsTRY, which exhibited the same expression pattern as *CsMYB6*. We also examined the molecular link between CsMYB6 and CsGL1 during trichome development.

## Materials and methods

### Plant material and growth conditions

Two monoecious cucumber inbred lines, R3407 [wild-type (WT)] and its ‘glabrous’ and wart-free *csgl1* mutant, were used in the present study. All plants were grown in a greenhouse under natural light in Beijing, China.

The Arabidopsis *nok* mutant (Col background) was obtained from The Arabidopsis Information Resource (http://www.arabidopsis.org/index.jsp; stock number SALK_025449) and Col was used as the WT control. Arabidopsis seeds were germinated on Murashige and Skoog (MS) medium containing 1% sucrose and 0.2% phytagar. Seeds were kept at 4 °C for 3 d and then were moved to 22 °C under a 16 h light/8 h dark regime. Seedlings were transferred to soil 7–10 d after germination.

### Sequence alignments and phylogenetic analysis

The coding sequence (CDS) of *CsMYB6* was amplified by PCR from female flower bud cDNA using gene-specific primers (Supplementary Table S1 at *JXB* online). The amino acid sequences of the related R2R3MYB proteins from *A. majus*, Arabidopsis, and cotton were obtained by BLAST searches of the National Center for Biotechnology Information nucleotide database (http://www.ncbi.nlm.nih.gov/nucleotide/) with the deduced amino acid sequence of CsMYB6. A multiple sequence alignment of CsMYB6 and the related R2R3MYB proteins was carried out as previously described (Zhang *et al.*, 2014). The phylogenetic tree was created with MEGA5 software, using the neighbor-joining method (Saitou and Nei, 1987) with 1000 bootstrap replicates.

### Spatial and temporal expression analysis by quantitative real-time RT–PCR

Total RNA was extracted from the roots, stems, male and female flower buds, and fruits of different stages using a Quick RNA isolation Kit (Huayueyang, China). cDNA was synthesized using a PrimeScript First Strand cDNA Synthesis Kit (TaKaRa). SYBR® Premix Ex Taq from TaKaRa was used for quantitative real-time reverse transcription–PCR (qRT–PCR) with an Applied Biosystems 7500 real-time PCR system (Applied Biosystems). The cucumber *α-TUBULIN (TUA*) gene was used as an internal control (Zhang *et al.*, 2014) in all qRT–PCRs. Three biological replicates and three technical replicates were performed to verify the accuracy of the expression data. The gene-specific qRT–PCR primers are listed in Supplementary Table S1.

### GUS expression and staining of transgenic cucumber plants

The putative promoter regions of *CsMYB6* and *CsTRY*, 2184 bp and 2000 bp fragments upstream of the start codon (ATG), respectively, were used to generate *pCsMYB6-GUS* and *pCsTRY-GUS* constructs, using gene-specific primers containing the *Pst*I (5ʹ end) and *Bam*HI (3ʹ end) sites, and the *Bam*HI (5ʹ end) and *Nco*I (3ʹ end) sites, respectively. The constructs were transformed into cucumber as described previously (Wang *et al.*, 2014). Histochemical staining for β-glucuronidase (GUS) activity was performed following Chen *et al.* (2016).

### RNA *in situ* hybridization

Female cucumber flower buds, carpopodium samples, and petioles were fixed, embedded, sectioned, and hybridized with digoxigenin-labeled probes as previously described (Zhang *et al.*, 2014). Digoxigenin-labeled sense and antisense RNA probes were obtained using the T7 and SP6 RNA polymerases (Roche). The primer pairs used are listed in Supplementary Table S1.

### Subcellular localization of *CsMYB6* and *CsTRY* in onion epidermal cells

The full-length coding regions of *CsMYB6* and *CsTRY* were cloned without the stop codon and inserted into the pEZS-NL vector (Chen *et al.*, 2016) between the *Xba*I and *Sma*I sites. An empty pEZS-NL vector was used as the positive control. Transient expression of the fusion proteins in onion epidermal cells was performed according to Varagona *et al.* (1992). An Olympus BX 51 fluorescence microscope was used to visualize the fluorescent signals.

### Scanning electron microscopy

Cucumber ovary samples were fixed, washed, postfixed, dehydrated, coated (Chen *et al.*, 2014), and observed using a Hitachi S-4700 scanning electron microscope with a 2 kV accelerating voltage.

### Ectopic expression of *CsMYB6* in Arabidopsis

To generate the *CsMYB6*-overexpressing lines, the full-length coding region of *CsMYB6* was amplified using specific primers containing the *Xba*I (5ʹ end) and *Sma*I (3ʹ end) endonuclease sites, and inserted in the reverse orientation into the pCAMBIA1305.1 vector (Chen *et al.*, 2016). The recombinant plasmids were transformed into Col (WT) and *nok* mutant plants using the floral dip method (Clough and Bent, 1998). The transgenic Arabidopsis plants were screened on MS medium with 25 mg l^−1^ hygromycin.

### Cucumber transformation

The full-length *CsTRY* coding region was amplified and inserted in the reverse orientation into the *Xba*I and *Sma*I sites of the pCAMBIA1305.1 vector (Chen *et al.*, 2016). The *CsMYB6* and *CsTRY* overexpression constructs were used for cucumber transformation. The recombinant plasmids were transformed into the cucumber R3407 (WT) line and *csgl1* mutant plants using a cotyledon transformation method as previously described (Wang *et al.*, 2014). The primers are listed in Supplementary Table S1.

### Dual-luciferase transient expression assay in tobacco leaves

The promoters of *pCsTRY* (1500 bp) were inserted into the vector pGreenII 0800-Luc (Hellens *et al.*, 2005) and the CDS of *CsMYB6* was inserted into pGreenII 62-SK (Hellens *et al.*, 2005). Tobacco (*Nicotiana benthamiana*) leaves were used for co-expression studies as previously described (Yotsui *et al.*, 2013). A no-effector construct was used as a negative control. The expression of firefly luciferase and renilla luciferase was examined using dual-luciferase assay reagents (Promega, USA). Data were collected as the ratio of firefly luciferase to renilla luciferase.

### Yeast one-hybrid assay

A yeast one-hybrid (Y1H) assay was performed using the Matchmaker Gold Yeast One-Hybrid System (Clontech). The three MYB binding site (MBS) motifs in the *CsTRY* promoter were cloned into the pAbAi vector. The full-length *CsTRY* coding region was amplified and inserted in the pGADT7 vector. The recombinant plasmids were transformed into the Y1H Gold strain. DNA–protein interaction was assessed based on the growth of the co-transformants on synthetic dextrose plates lacking leucine, but containing aureobasidin A. The primers are listed in Supplementary Table S1.

### Electrophoretic mobility shift assay

The full-length CsMYB6 protein sequence was fused in frame with a GST tag and expressed in *Escherichia coli* BL21 cells by the addition of isopropyl β-D-1-thiogalactopyranoside to a final concentration of 1 mM, before the cultures were incubated at 18 °C for 16 h. The recombinant protein was purified by using glutathione Sepharose 4B beads (GE Healthcare). An electrophoretic mobility shift assay (EMSA) was performed using the Light Shift Chemiluminescent EMSA kit (Thermo Fisher Scientific, Shanghai, China) according to the manufacturer’s instructions. The biotin-labeled DNA fragments were used as probes, while unlabeled fragments of the same sequence were used as the competitors.

### *CsMYB6* transcriptional activation analysis

The nuclear localization signal (NLS) and GAL4 AD sequences were amplified from the pGADT7 vector (Clontech) by PCR and ligated into the pGBKT7 vector (Clontech) fused to the GAL4 DNA BD. The fusion vector, pGBKT7-NLSAD, was used as a positive control and the pGBKT7 vector was used as a negative control. To determine the transcriptional activation portion of CsMYB6, the full-length or partial CDS [corresponding to deletions of amino acids 1–11 (ΔN1-11), 1–64 (ΔN1-64), or 1–128 (ΔN1-128) at the N-terminus; 12–234 (ΔC12-234) or 129–234 (ΔC129-234) at the C-terminus; and ΔN1-11, 129-234 (ΔN1-11, ΔC129-234)] of *CsMYB6* were fused to the BD in the pGBKT7 vector. The recombinant constructs were separately transformed into yeast strain AH109. The transformed yeast cells were grown on synthetic defined (SD) plates lacking tryptophan and histidine (SD/-Trp-His) and lacking tryptophan, histidine, and adenine (SD/-Trp-His-Ade) with α-gal, and on control plates lacking only tryptophan (SD/-Trp). All experiments were performed according to the manufacturer’s user manual (Clontech).

### Yeast two-hybrid assay

A yeast two-hybrid (Y2H) library derived from the RNA from –7 days post anthesis (DPA) to 4 DPA cucumber ovaries was constructed using a commercial service (Oebiotech; Shanghai, China). The library was screened using the truncated C-terminal CsMYB6 protein (N65-234) as the bait according to the manufacturer’s instructions (Clontech). For the yeast two-hybrid assay, we cloned the cDNA sequence of *CsTRY* (full-length) and fused it into the pGBKT7 and pGADT7 vectors, respectively. The combination of TTG1-AD and GL1-BD was used as a positive control (Chen *et al.*, 2016). All recombinant constructs were separately transformed into the yeast strain AH109. The growth conditions of the yeast cells were as described above.

### Bimolecular fluorescence complementation assay

To generate the bimolecular fluorescence complementation (BiFC) constructs, the full-length cDNA sequences of *CsTRY* and *CsMYB6* were cloned and fused with the pSPYCE and pSPYNE vectors (Walter *et al.*, 2004). Tobacco (*N. benthamiana*) leaves were used for co-expression studies as previously described (Schütze *et al.*, 2009). The fluorescence signal was detected 2 to 4 d after infiltration, using an Olympus BX 51 fluorescence microscope to acquire fluorescent images. Yellow fluorescent protein (YFP) imaging was performed at an excitation wavelength of 488 nm.

### Co-immunoprecipitation assay

Tobacco (*N. benthamiana*) leaves were infiltrated with *Agrobacterium* strain GV3101 harboring CsMYB6-6×His and CsTRY-HA. Three days later, tobacco leaves were collected and total proteins were extracted from them. CsMYB6-6×His and CsTRY-HA proteins were immunoprecipitated by anti-HA and anti-His agarose conjugate (Sigma) separately. Proteins bound to the beads were resolved by lysis buffer and dissolved with 2× loading buffer. After being boiled, the samples were resolved via sodium dodecyl sulfate polyacrylamide gel electrophoresis and transferred on to nitrocellulose membranes (Millipore). The membranes were incubated with anti-HA and anti-His at 4 °C.

### Accession numbers

GenBank accession numbers of R2R3MYB protein sequences used in this study included Cucumber CsMYB26 (Csa009688); snapdragon AmMYBML1 (CAB43399), AmMYBML2 (AAV70655), AmMYBML3 (AAU13905), and AmMIXTA (CAA55725); Petunia PhMYB1 (CAA78386); Arabidopsis AtMYB0 (GL1) (NP_189430), AtMYB5 (NP_187963.1), AtMYB23 (NP_198849.1), AtMYB16 (NP_197035), AtMYB17 (NP_191684), AtMYB106 (NP_186763), and AtMYB66(Wer) (NP_196979); cotton GaMYB2 (AAU12248), GhMYB109 (CAD71140) GhMYB25 (AAK19616), and GhMYB25-like (EST AY464066); and tomato ShMYB1 (EST241733).

## Results

### *CsMYB6* is a homolog of Arabidopsis MIXTA transcription factor

**Fig. 1.**
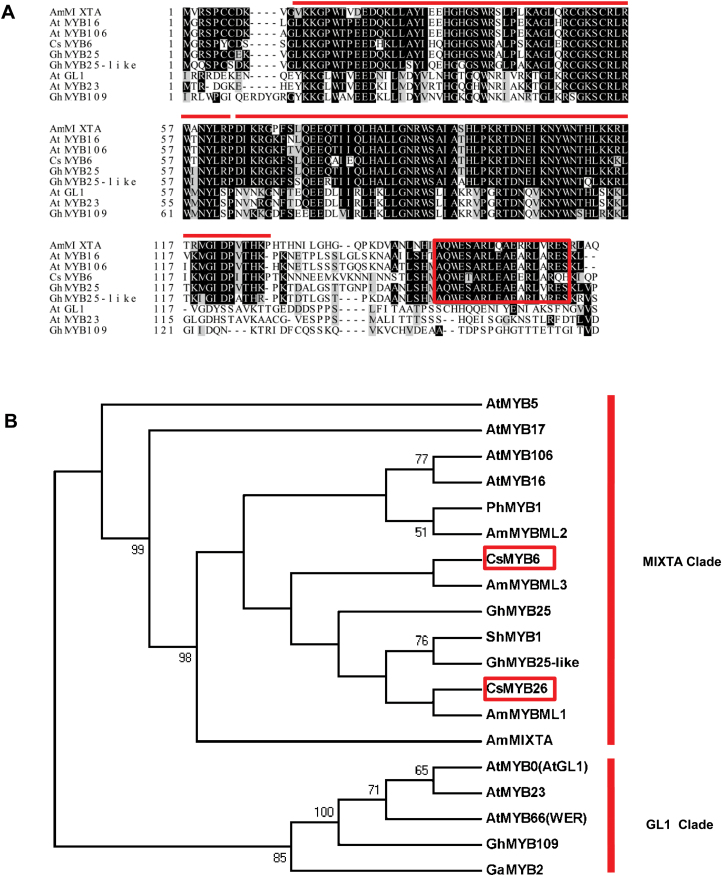
Phylogenetic analyses of *CsMYB6* and its homologs in various species. (A) Protein sequence alignment of MYB6 homologs from Arabidopsis (AtMYB106, AtMYB16, AtGL1, AtMYB23), snapdragon (AmMIXTA), cotton (GhMYB109, GhMYB25, GhMYB25-like), and cucumber (CsMYB6). The lines above the sequence indicate the conserved R2 and R3 MYB repeats. The box indicates the conserved MIXTA motif in subgroup 9 of R2R3MYB (AQWESARXXAEXRLXRES). (B) Phylogram of CsMYB6, CsMYB26, and related R2R3MYB proteins from Arabidopsis, snapdragon, Petunia, tomato, cotton, and cucumber. The number at each node is the probability supporting each node with 1000 bootstrapping values.

Alignment of CsMYB6 and its homologs from Arabidopsis (AtMYB106, AtMYB16, AtGL1, AtMYB23), snapdragon (AmMIXTA), and cotton (GhMYB109, GhMYB25, GhMYB25-like) revealed that the CsMYB6 protein shared the conserved R2R3MYB repeat region and amino acid sequence of R2R3MYB subgroup 9, and had the highest similarity with cotton GhMYB25, followed by AtMYB106 and AtMYB16. CsMYB6 was more distantly related to AtGL1, AtMYB23, and cotton GhMYB109 (Fig. 1A). A phylogenetic analysis of the cucumber MYB6 protein and the R2R3MYB proteins from Arabidopsis, snapdragon, petunia, tomato, and cotton, which have been shown to be involved in trichome development (Perez-Rodriguez *et al.*, 2005; Jaffé *et al.*, 2007; Jakoby *et al.*, 2008; Pu *et al.*, 2008; Li *et al.*, 2012; Yoshimi *et al.*, 2013), showed that it clustered within the MIXTA clade, which includes other known MIXTA-like transcription factors, such as AmMIXTA, AmMYBML1, GhMYB25, and GhMYB25-like, and was distinct from the MYBs, such as AtGL1, AtWER, and the cotton GL1-like MYB GhMYB109 (Fig. 1B). Expression analysis with qRT-PCR confirmed lower expression of *CsMYB6* in various organs of the *csgl1* mutant than in the WT line (Supplementary Fig. S1). These observations indicated that *CsMYB6* may be involved in trichome development in cucumber.

### *CsMYB6* is highly expressed in cucumber ovary epidermal cells

**Fig. 2.**
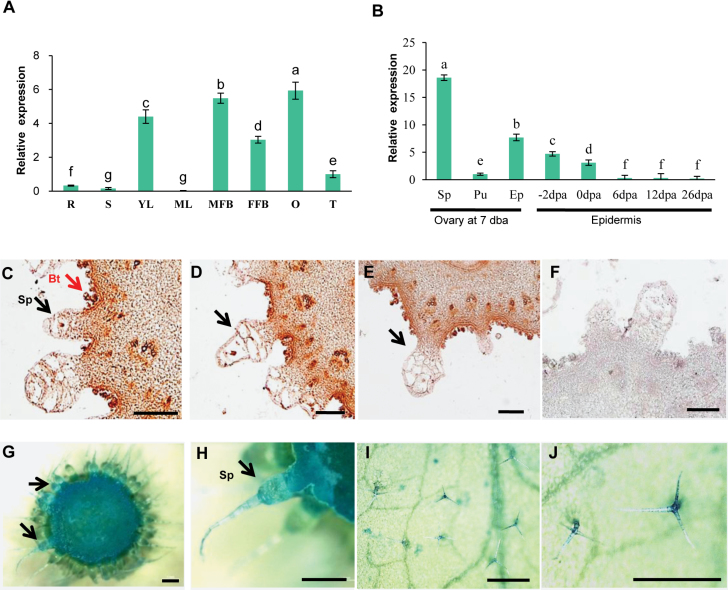
Spatial-temporal expression patterns of *CsMYB6*. (A) *CsMYB6* expression in different organs based on the results of qRT–PCR. (B) *CsMYB6* expression in different parts and developmental stages of ovaries. The cucumber gene *α-TUBULIN* (*TUA*) was used as an internal control. Significant differences were determined according to Duncan’s multiple range test (*P*<0.05). Error bars represent the SD based on three biological replicates. (C–E) mRNA in situ hybridization of *CsMYB6* in cucumber ovaries at 7 days before anthesis (DBA). Strong signals are detected in spines, bloom trichomes, epidermis, and pulp adjacent to the epidermis. Arrows indicate the locations of expression of *CsMYB6* in fruit trichomes. (F) Negative control using the sense probe at 7 DBA. (G–J) GUS expression patterns in *pCsMYB6-GUS* transgenic lines. GUS staining in trichomes from cucumber ovaries (G, H) and Arabidopsis leaves (I, J). Bt, bloom trichome; DPA, days post anthesis; Ep, epidermis including fine spines; FFB, female flower bud; MFB, male flower bud; O, ovary (at 7 DBA); Pu, pulp; R, Root; S, stem; Sp, spines; T, tendril; YL, young leaf. Scale bars=200 µm in C–F, 1 mm in G and H, and 500 µm in I and J.

To better understand the biological function of *CsMYB6*, we evaluated its expression in various organs (roots, stems, young leaves, mature leaves, female flower buds, male flower buds, fruits, and tendrils) using qRT–PCR. The highest expression of *CsMYB6* occurred in young leaves and both male and female flower buds (Fig. 2A). We analyzed *CsMYB6* transcript levels in different parts of the cucumber ovary at 7 days before anthesis (DBA; the stage of fruit spine initiation and development); higher expression was detected in the epidermis (including peel and spines) than in the pulp (Fig. 2B). Transcript levels were also assessed during different cucumber fruit developmental stages. *CsMYB6* was abundantly expressed in the peel at the stage of fruit spine initiation (7 DBA), but expression declined rapidly as the spines began to elongate (Fig. 2B). When the expression patterns of *CsMYB6* in 7 DBA ovaries were analyzed by *in situ* hybridization, *CsMYB6* transcripts were detected in spines, tubercules, bloom trichomes, the epidermis, and the pulp adjacent to the epidermis, which was consistent with the qRT–PCR results (Fig. 2C–E). In addition, these results were supported by *CsMYB6* promoter GUS reporter gene analysis, in which *pCsMYB6-GUS*-expressing transgenic cucumber lines showed GUS activity mainly in the spines, tubercules, epidermis, and pulp, and *pCsMYB6-GUS*-expressing Arabidopsis lines showed GUS activity mainly in the trichomes (Fig. 2G–I). These observations suggested that *CsMYB6* may be involved in epidermal cell differentiation and fruit trichome initiation in cucumber.

### Overexpression of *CsMYB6* reduces cucumber trichome density

**Fig. 3.**
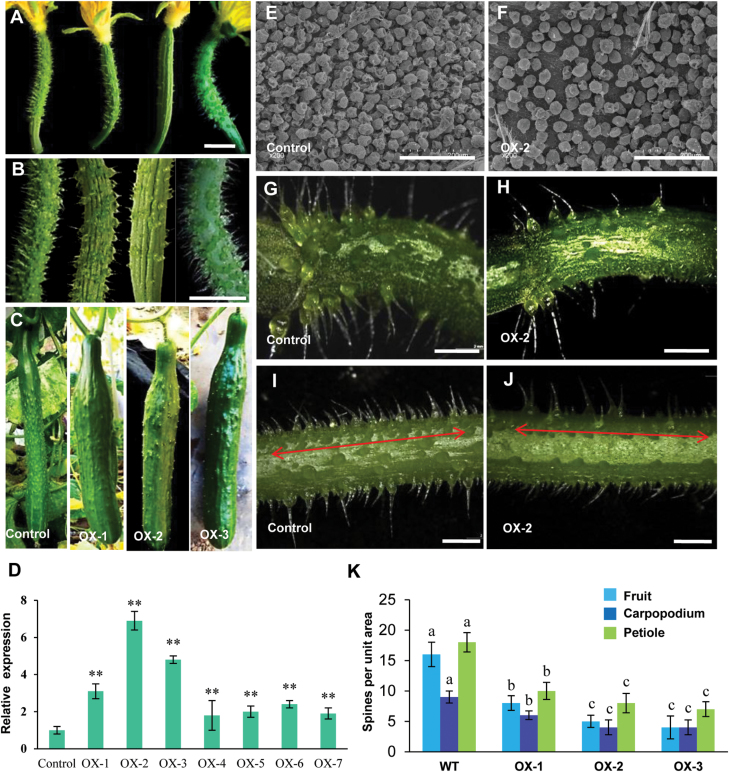
Phenotypes and gene expression analysis of *35S:CsMYB6* transgenic cucumber plants. Appearance of female flowers of different *35S:CsMYB6* lines (A) and fruits at 2 (B) or 12 (C) days post anthesis. (D) qRT–PCR analyses of *CsMYB6* in wild-type (WT) plants and transgenic overexpression (OX) lines. The cucumber gene *α-TUBULIN* (*TUA*) was used as an internal control. Error bars represent the SD of three biological replicates. (E, F) Scanning electron microscopic images of the fruit surface of a WT plant (E) and a *35S:CsMYB6*-overexpressing line (F) at anthesis. (G, H) Carpopodium of a WT plant (G) and the *35S:CsMYB6*-overexpressing line OX-2 (H). (I, J) Petiole of a WT plant (I) and the *35S:CsMYB6*-overexpressing line OX-2 (J). (K) Number of trichomes in WT and *35S:CsMYB6*-overexpressing lines. Error bars represent the SE. Significant differences were determined according to Duncan’s multiple range test (*P*<0.05) or Student’s *t*-test (*P<0.05, **P<0.01). Scale bars=0.5 cm in A and B, 200 μm in E and F, and 2 mm in G–J.

In order to verify the function of *CsMYB6* in cucumber, the *35S:CsMYB6* overexpression vector was introduced into the cucumber inbred line R3407 by *Agrobacterium*-mediated cotyledon transformation. Antibiotic selection and genomic PCR were used to screen transgenic plants (Zhang *et al.*, 2014; Cheng *et al.*, 2015; Chen *et al.*, 2016). Seven positive overexpression lines were obtained (Fig. 3D). Three representative overexpression lines (OX-1, OX-2, and OX-3), whose *CsMYB6* expression levels were much higher than the levels in the WT line, were selected for detailed studies. We observed that the numbers of trichomes on the fruit surface, carpopodium, and petiole were substantially lower in all three lines compared with the WT (Fig. 3A–C, G–J), and the numbers of spines on fruits at 0 d post pollination were 50%, 75%, and 69% lower in line OX-1, OX-2, and OX-3, respectively, than in WT plants (Fig. 3I). Moreover, there were substantially fewer bloom trichomes on the surface of the fruit of the transgenic lines than on WT plants (Fig. 3E, F). There were no obvious differences between the overexpression lines and WT plants in terms of spine appearance or morphological characteristics (Supplementary Fig. S2).

### *CsMYB6* suppresses fruit trichome initiation in the *csgl1* mutant

**Fig. 4.**
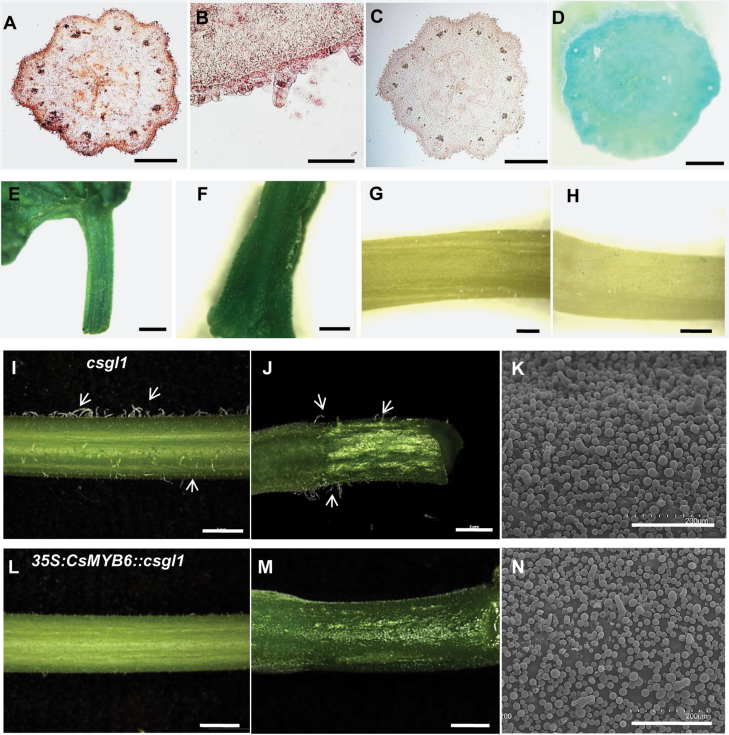
Expression pattern and functional analysis of *CsMYB6* in the *csgl1* mutant. (A, B) mRNA *in situ* hybridization of *CsMYB6* in ovaries from the *csgl1* mutant at 7 days before anthesis (DBA). A strong signal was detected in the epidermis (A) and trichome (B). (C) Negative control using the sense probe at 7 DBA. (D–F) GUS expression patterns in ovaries (D), petiole (E), and carpopodium (F) from the *pCsMYB6-GUS* transgenic *csgl1* plants. (G, H) Negative control of GUS staining in petiole and carpopodium from the *csgl1* mutant. (I–N) Phenotype of the *csgl1* mutant (I–K) and the *CsMYB6*-overexpressing *csgl1* mutant (L–N). Morphological observations of petiole (I, L) and carpopodium (J, M), and scanning electron microscopic images of the fruit surface (K, N). Overexpression of *CsMYB6* resulted in a lower density of trichomes on the fruit surface, carpopodium, and petiole than in the *csgl1* mutant. Scale bars=500 µm in A and C, 50 µm in B, 1 mm in D–H, 2 mm in I, J, L, and M, and 200 µm in K and N.

The spontaneous *csgl1* (*mict*) glabrous cucumber mutant has fruit trichomes with a massive reduction in size and a stunted morphology (Zhao *et al.*, 2015). However, further characterization of *csgl1* revealed many obvious macro-size trichomes on the epidermis of the carpopodium and petiole (Fig. 4I, J). In a previous study, the expression of *CsMYB6* in the *csgl1* mutant was found to be reduced to 11% of that in the WT (Li *et al.*, 2015). We characterized the expression of *CsMYB6* in the *csgl1* mutant using RNA *in situ* hybridization and a promoter::GUS assay, and found that *CsMYB6* was still expressed in the peel, carpopodium, and petiole (Fig. 4A, B, D–F). To understand the function of *CsMYB6* in fruit trichome formation in the *csgl1* mutant, the *CsMYB6* gene was overexpressed in the *csgl1* mutant. We found that overexpression of *CsMYB6* reduced trichome density on the fruit surface, carpopodium, and petiole compared with the *csgl1* mutant; however, the abnormal morphologic phenotype of the trichomes was not rescued (Fig. 4I–N). These results suggested that *CsMYB6* suppresses the initiation of fruit trichomes in the *csgl1* mutant.

To further verify the function of *CsMYB6* in regulating the initiation of spines, the *CsMYB6-RNAi* construct was introduced into the WT line and the *csgl1* mutant. However, phenotypic analysis of *CsMYB6*-*RNAi* transgenic cucumber plants showed no difference in trichome morphology (Supplementary Fig. S3).

### *CsTRY* exhibits a similar expression pattern to *CsMYB6* and overexpression of *CsTRY* reduces trichome numbers on fruits and petioles

**Fig. 5.**
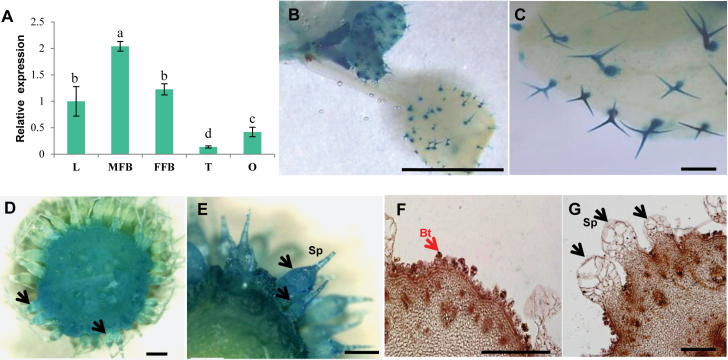
Expression analysis of *CsTRY*. (A) qRT–PCR analysis of *CsTRY* expression in different organs of cucumber. The cucumber gene *α-TUBULIN* (*TUA*) was used as the internal control. Significant differences were determined according to Duncan’s multiple range test (*P*<0.05). Error bars represent the SD of three biological replicates. (B–E) GUS expression patterns in *pCsTRY-GUS* transgenic lines. GUS staining in Arabidopsis leaf trichomes (B, C) and cucumber ovaries (D, E). (F, G) mRNA *in situ* hybridization of *CsTRY* in cucumber ovaries at 7 days before anthesis (DBA). FFB, female flower bud; L, leaf; MFB, male flower bud; O, ovary at 7 DBA; T, tendril. Scale bars=2 mm in B, 200 µm in C, F, and G, and 1 mm in D and E.

Our previous study found that *CsTRY* was the only cucumber homolog of Arabidopsis *AtTRY*, *AtETC1*, and *AtCPC*, and that, additionally, ectopic overexpression of *CsTRY* in WT Arabidopsis significantly reduced the number of leaf trichomes (Tan *et al.*, 2012). We hypothesize that *CsTRY* may perform a similar function to *CsMYB6* in cucumber trichome development. We examined the expression pattern of *CsTRY* in leaves, female and male flower buds, ovaries, and tendrils using qRT–PCR. We observed that *CsTRY* was mainly expressed in leaves, male and female flower buds, and ovaries, which was consistent with the expression pattern of *CsMYB6* (Fig. 5A). Furthermore, in the *pCsTRY::GUS* assay, *pCsTRY-GUS*-expressing Arabidopsis lines showed GUS activity mainly in the trichomes (Fig. 5B, C), while GUS expression in *pCsTRY-GUS*-expressing transgenic cucumber lines occurred in the trichomes, epidermis, and pulp adjacent to the epidermis of the ovaries (Fig. 5D, E). We examined the expression pattern of *CsTRY* in 7 DBA ovaries by using RNA *in situ* hybridization; results were consistent with those of the *pCsTRY::GUS* assay, indicating that *CsTRY* transcripts were expressed in spines, tubercules, epidermis, and pulp adjacent to the epidermis (Fig. 5F, G). These data suggested that *CsTRY* may be involved in fruit trichome development in cucumber.

**Fig. 6.**
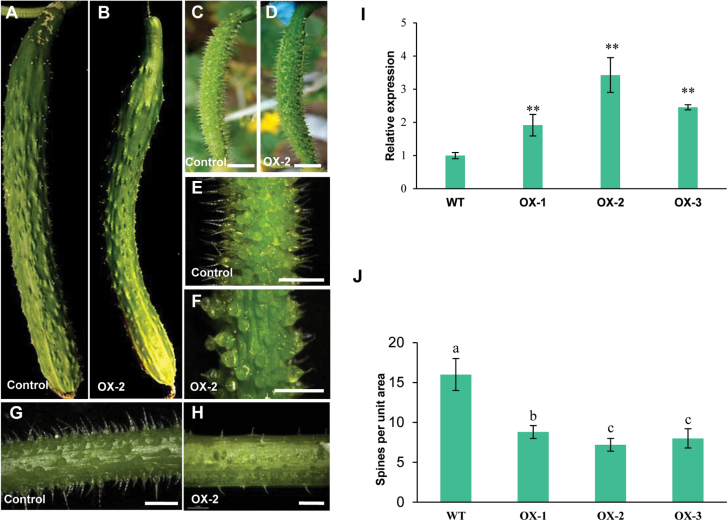
Overexpression of *CsTRY* results in fewer trichomes on cucumber fruit. The fruit spine numbers of the *35S:CsTRY*-overexpressing line OX-2 (B, D, F) were smaller than those of the wild type (WT) (A, C, E); this was also the case for the petiole of the WT (G) and the OX-2 line (H). (I) qRT–PCR analyses of *CsTRY* in WT plants and three transgenic *35S:CsTRY*-overexpressing lines. The cucumber gene *α-TUBULIN* (*TUA*) was used as the internal control. Error bars represent the SD of three biological replicates. (J) The number of trichomes in WT and *35S:CsTRY*-overexpressing lines. Significant differences were determined according to Duncan’s multiple range test (*P*<0.05) or Student’s *t*-test (**P<0.01). Error bars represent the SE. Scale bars=0.5 cm in C–F and 2 mm in G and H.

The *CsTRY* coding sequence (*CsTRY-OE*) was fused to the CaMV 35S promoter and introduced into the cucumber inbred line R3407. Five positive overexpression lines were obtained (Fig. 6I). We found that the numbers of trichomes on the fruits and petioles of all transgenic lines were lower than those in the WT line (Fig. 6A–H). Consistent with this observation, the numbers of fruit spines at 0 d post pollination in three overexpressing lines (OX-1, OX-2, and OX-3) were 38%, 44%, and 41% of those in the WT, respectively (Fig. 6J), suggesting that *CsTRY* may suppresses the formation of trichomes in cucumber fruits, similar to *CsMYB6*.

### *CsTRY* is directly regulated by *CsMYB6*

**Fig. 7.**
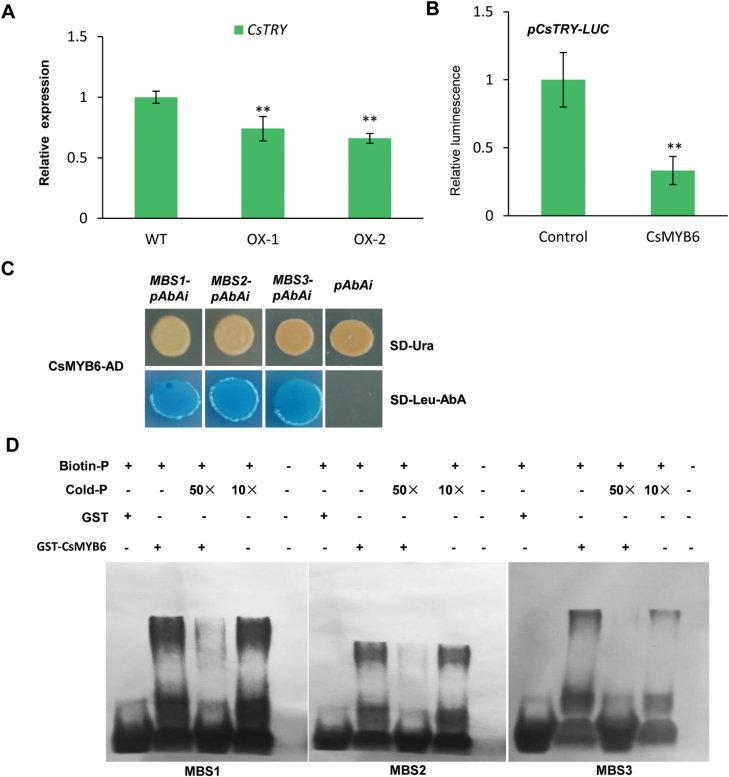
(A) Gene expression changes of *CsTRY* in *CsMYB6*-overexpressing cucumber plants. Asterisks indicate statistically significant differences according to Student’s *t*-test (**P<0.01) WT, wild type. (B) Transient transcriptional activity assay showing the repression of the *CsTRY* promoter by CsMYB6. Asterisks indicate statistically significant differences according to Student’s *t*-test (**P<0.01). (C) Transactivation activity of CsMYB6 to the three MBS motifs in the promoter of *CsTRY* in yeast. (D) An electrophoretic mobility shift assay was used to analyze the interaction of GST-CsMYB6 and the three MBS motifs in the promoter of *CsTRY*. Purified GST-CsMYB6 protein samples (3 µg) were incubated with 25 pM of the biotin-labeled WT probe. Non-labeled probes at 50-fold concentrations were added for the competition test.

To further reveal the links between *CsTRY* and *CsMYB*6 in cucumber trichome development, we examined the expression of *CsTRY* in *CsMYB6*-overexpressing cucumber lines, and found significantly lower expression of *CsTRY* in the peel of the transgenic *CsMYB6*-overexpressing plants (Fig. 7A). The dual-luciferase assay also showed that CsMYB6 attenuated the promoter activity of *CsTRY* in *N. benthamiana* leaves (Fig. 7B).This observation and the fact that three MYB binding sites are present in the *CsTRY* promoter region suggested the possibility that CsMYB6 may directly bind to the *CsTRY* promoter. To test this hypothesis, we performed Y1H assays and found that CsMYB6 bound to all the three MBS elements in the *CsTRY* promoter (Fig. 7C); this finding was further confirmed with EMSA (Fig. 7D).

**Fig. 8.**
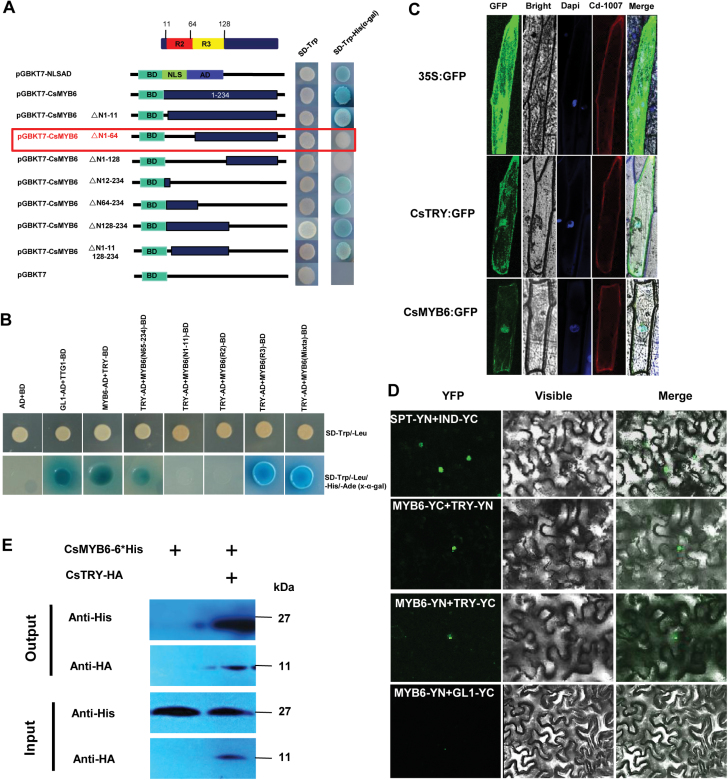
CsMYB6 interacts with CsTRY. (A) Deletion analysis for screening the transcriptional activation regions of CsMYB6. Different parts of CsMYB6 were fused with the GAL4 DNA-binding domain and transformed into yeast strain AH109 containing the *His3* and *LacZ* reporter genes. Three repeats were performed and each showed similar patterns. AD, GAL4 activation domain; BD, GAL4 DNA-binding domain; NLS, nuclear localization signal. (B) Yeast two-hybrid assay showing that the R3 and MIXTA domains of CsMYB6 interacted with CsTRY by growth on SD/-Leu/-Trp/-His/X-α-gal plates. The empty vector was used as a negative control, and the interaction between TTG1-BD and GL1-AD was used as a positive control. (C) Subcellular localization of CsTRY and CsMYB6 protein in onion epidermal cells. (D) BiFC analysis of the physical interaction between CsMYB6 (fused with the N-terminal or C-terminal fragment of YFP) and CsTRY (fused with the C-terminal or N-terminal fragment of YFP). INDEHISCENT (IND)-YFPC and SPATULA (SPT)-YFPN were used as positive controls. Different combinations of the fused constructs were co-expressed in leaves of *N. benthamiana*, and the cells were then visualized using confocal microscopy. (E) Co-immunoprecipitation of transiently co-expressed CsMYB6-6×His and HA-CsTRY in *N. benthamiana* leaves. Protein extracts before (input) and after (output) immunoprecipitation with anti-His antibody-conjugated beads were detected by western blot with anti-HA antibody.

To better understand the regulatory mechanism of *CsMYB6*, Y2H assays were used to screen interaction partners of CsMYB6 in the cucumber genome. Because of the strong transcriptional self-activation of CsMYB6, a deletion analysis was performed; we found that the activation domain (AD) was localized in residues 1–64 at the N-terminus (Fig. 8A). Consequently, a Y2H assay was performed using the truncated C-terminal protein containing amino acids 65–234 of CsMYB6 as the bait protein to screen a cucumber cDNA library fused to the yeast GAL4 AD. Eighteen putative interacting proteins were identified, which included, unexpectedly, CsTRY (Supplementary Table S2). Additional Y2H assays indicated that CsTRY interacted directly with the R3 and MIXTA domains of CsMYB6 (Fig. 8B). Subcellular localization of CsMYB6 and CsTRY in onion epidermal cells revealed that both proteins were localized mainly to the nucleus and plasma membrane (Fig. 8C). In further BiFC and co-immunoprecipitation assays, the interaction between CsMYB6 and CsTRY was proven *in planta* (Fig. 8D, E). Therefore, all these data supported the existence of direct interactions between CsMYB6 and CsTRY.

### Overexpression of *CsMYB6* in Arabidopsis results in fewer trichomes

**Fig. 9.**
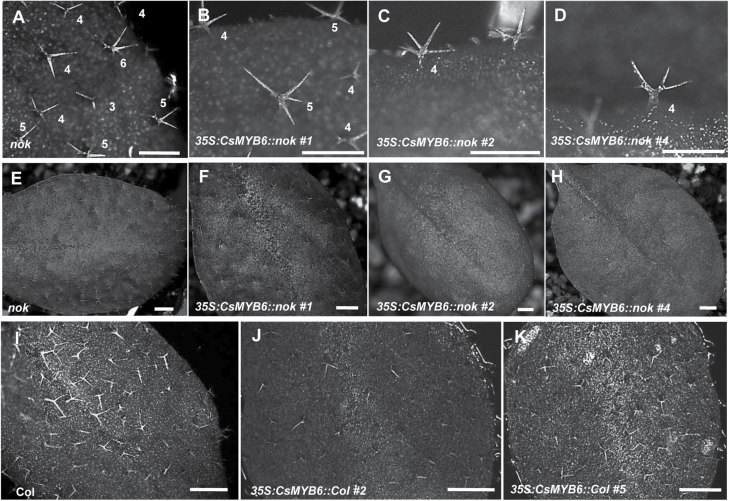
Ectopic expression of *CsMYB6* in the *nok* mutant and wild-type Arabidopsis. (A–K) Phenotypic comparison of leaf trichomes from *nok* (A, E), 35S::*CsMYB6/nok* lines (B–D, F–H), Col (I), and 35S:*CsMYB6*/Col lines (J, K) Arabidopsis plants. Overexpression of *CsMYB6* in *nok* and Col both resulted in fewer trichomes. Scale bars=1 mm in A–D and 2 mm in E–K.

To study the extent to which *CsMYB6* function is conserved among core eudicots, we tested the ability of *CsMYB6* to recover the mutant phenotype of *nok/myb106* (Fig. 9A; Folkers *et al.*, 1997). The 35S promoter was used instead of the *AtMYB106* promoter because *AtMYB106* shows a different expression pattern from *CsMYB6* (Jakoby *et al.*, 2008). Six independent transgenic plants were obtained, all of which showed partial rescued of the over-branched trichome phenotype of the *nok* mutant (Fig. 9B–D, Supplementary Table S3). All the overexpression lines had a 10–20% higher proportion of trichomes with four branches than the *nok* mutant, while the number of trichomes with five branches in the overexpression lines decreased by 10–15% (Supplementary Table S3). Interestingly, the total number of trichomes on these transgenic plants was reduced compared with the *nok* mutant (Fig. 9E–H). For example, plants from transgenic line 4 had 41% fewer trichomes than the *nok* plants (Supplementary Table S3).

To further examine the function of *CsMYB6*, we overexpressed *CsMYB6* in WT Arabidopsis (Col). In all six independent transgenic lines obtained, the number of trichomes was lower than in WT plants (Fig. 9I–K). Moreover, in *35S:CsMYB6* lines, trichomes exhibiting two branches emerged, which were similar to the trichomes of *AtMYB106/NOK*-overexpressing Arabidopsis plants (Supplementary Fig. S4). These results suggested that *CsMYB6* also has a suppressive role in the regulation of trichome development in Arabidopsis.

## Discussion

Trichomes are specialized epidermal cells that are located in the aerial parts of plants. Several MYB transcription factors have been identified that are involved in the regulation of epidermal cell patterning in Arabidopsis and cotton, species in which trichomes are unicellular structures (Loguerico *et al.*, 1999; Suo *et al.*, 2003; Wilkins and Arpat, 2005; Lee *et al.*, 2006; Lee *et al.*, 2007; Wu *et al.*, 2006). Little is known about the function of MYB transcription factors in the development of cucumber multicellular trichomes. In this study, we provide evidence to show that the R2R3MYB transcription factor gene *CsMYB6* plays a key role in cucumber fruit trichome formation. We found that, unlike the function of its homologs in Arabidopsis and cotton, both of which have single-celled trichomes, CsMYB6 negatively regulates fruit trichome formation through an interaction with CsTRY, which is independent of CsGL1.

### *CsMYB6* is a MIXTA-like gene of the R2R3MYB family

Since the first cloning of the *MIXTA* R2R3MYB transcription factor gene in snapdragon (Noda *et al.*, 1994), MIXTA homologs have been identified in many plant species, such as *Thalictrum thalictroides*, *Erythranthe lewisii*, tomato (*Solanum lycopersicum*), cotton, and Arabidopsis. MIXTA homologs have been shown to regulate epidermal cell morphology, including the differentiation of trichome cells (e.g. Glover *et al.*, 1998; Perez-Rodriguez *et al.*, 2005; Jaffé *et al.*, 2007; Baumann *et al.*, 2007; Yoshimi *et al.*, 2013). Here, we studied CsMYB6, a MIXTA homolog in cucumber (Fig. 1). Protein sequence alignment among MIXTA homologs of different species revealed that the CsMYB6 protein shared the conserved R2R3MYB repeat region and amino acid sequence of R2R3MYB subgroup 9 (Fig. 1A). Overexpression of *CsMYB6* reduced trichome density in both cucumber and Arabidopsis. These data provided convincing evidence for a role of *CsMYB6*, like other MIXTA-like MYBS, in regulating epidermal cell differentiation and fruit trichome formation (Figs 3, 9). Phylogenetic analysis revealed that CsMYB6 was in the MIXTA clade, which also included Arabidopsis AtMYB106 and cotton GhMYB25-like (Fig. 1B). However, *AtMYB106*-overexpressing Arabidopsis plants showed trichomes that were less branched, suggesting that *AtMYB106* suppresses the formation of trichome branching, rather than trichome formation itself (Folkers *et al.*, 1997; Jakoby *et al.*, 2008). Unlike *AtMYB106*, *GhMYB25-like* is a key regulator in cotton fiber initiation (Walford *et al.*, 2011). In the present study, overexpression of *CsMYB6* affected the number of trichomes rather than their morphology, a function more like that of the cotton *GhMYB25-like* gene. These observations suggested that the role of *CsMYB6* in trichome development is more similar to that of *GhMYB25-like* than *AtMYB106.*

### *CsMYB6* is involved in the regulation of fruit trichome initiation in cucumber

In this study, expression analysis revealed spatial-temporal dynamics of *CsMYB6* expression in cucumber. We found that *CsMYB6* transcript was more abundant in ovaries at the stage of fruit spine initiation and development, and declined rapidly as the spines began to elongate (Fig. 2B). Furthermore, *CsMYB6* was expressed at a much higher level in the epidermis than in the pulp; both *in situ* hybridization and pCsMYB6::GUS assays clearly showed that *CsMYB6* was expressed in the fruit epidermis and trichomes (Fig. 2C–J). These data are consistent with the role of *CsMYB6* in regulating epidermal cell differentiation and fruit trichome formation. Since the overexpression of *CsMYB6* in both WT and *csgl1* mutant cucumber lines caused a significant decrease in trichome numbers in the fruit, petiole, and carpopodium (Figs 3, 4), *CsMYB6* may suppress fruit trichome initiation. This was further supported by ectopic expression of *CsMYB6* in Arabidopsis WT (Col) and *nok* (*AtMYB106*) mutant lines, which resulted in a reduced number of trichomes (Fig. 9). However, altered trichome morphology was observed in transgenic Arabidopsis plants overexpressing *CsMYB6*; this observation may have two possible explanations. Unlike the simple unicellular, highly branched trichomes of Arabidopsis, cucumber trichomes are multicellular and unbranched. Therefore, *CsMYB6* does not seem to be involved in trichome branching. In addition, the regulatory mechanisms for trichome formation in cucumber may differ from those in Arabidopsis, and *CsMYB6* and *AtMYB106* may play distinct roles in this process.

Phenotypic analysis of *CsMYB6*-*RNAi-WT* and *CsMYB6*-*RNAi-csgl1* transgenic cucumber plants showed no difference in the number and morphology of trichomes compared with their respective controls (Supplementary Fig. S3). The expression of *CsMYB26* was increased in *CsMYB6-RNAi* lines, and the phylogenetic analysis of CsMYB6 and CsMYB26 showed that they both clustered within the MIXTA clade (Fig. 1), indicating that CsMYB26 might have some functional redundancy with CsMYB6.

### *CsMYB6* acts upstream of *CsTRY* to regulate fruit trichome formation in cucumber

In Arabidopsis, AtTRY suppresses the formation of trichomes through interaction with GL3/EGL3 in competition with GL1 (Schellmann *et al.*, 2002; Esch *et al.*, 2003; Zhang *et al.*, 2003). The SQUAMOSA PROMOTER BINDING PROTEIN LIKE 9 transcription factors suppress trichome formation by directly binding to the *TRY* gene promoter and activating its expression (Nan *et al.*, 2010). The WRKY protein TTG2 binds to the promoter of TRY and regulates *TRY* expression through the enhancement of activator complex-triggered activation (Pesch *et al.*, 2014). *TRY* appears to play an essential role in the gene regulatory network underlying trichome patterning. Previous studies suggested that *CsTRY* is the only putative cucumber homolog of the Arabidopsis genes *AtTRY, AtETC1*, and *AtCPC*, and ectopic overexpression of *CsTRY* in Arabidopsis WT plants significantly reduced the number of leaf trichomes, indicating that *CsTRY* may have the same function as *AtTRY* (Tan *et al.*, 2012). Here, we generated *CsTRY*-overexpressing transgenic cucumber lines, which exhibited significantly reduced trichome initiation on the fruit (Fig. 6J). Thus, it is reasonable to conclude that, similar to its Arabidopsis homolog *AtTRY*, *CsTRY* suppresses fruit trichome initiation in cucumber. *CsTRY* was predominantly expressed in the trichomes, epidermis, and pulp adjacent to the ovary epidermis, showing overlapping expression with *CsMYB6*. In *35S:CsMYB6* plants, the *CsTRY* transcript level was lower than in WT plants (Fig. 7A). The dual-luciferase assay also showed that CsMYB6 can attenuate the promoter activity of *CsTRY* in *N. benthamiana* leaves (Fig. 7B). In further Y1H and EMSA assays, CsMYB6 was able to bind to the promoter of *CsTRY* (Fig. 7C, D). From these data, it seems that there is a novel mechanism in which a *CsMYB6-CsTRY* complex negatively regulates fruit trichome formation in cucumber. *CsMYB6* acted upstream of *CsTRY* in this process. Interestingly, we identified that overexpression of *CsMYB6* or *CsTRY* results in fewer trichomes. However, overexpression of *CsMYB6* inhibited, rather than promoted, the expression of *CsTRY*. Thus, the relationship between CsMYB6 and CsTRY seems not to be simple, and the details of the relationship remain to be identified. In Arabidopsis, AtGL3, AtTTG1, and AtGL1 were found to compose a trimeric complex and to activate trichome initiation (Oppenheimer *et al.*, 1991; Walker *et al.*, 1999; Zhang *et al.*, 2003). AtGL3 and AtTTG1 can enhance the activation of the *AtTRY* promoter, while AtGL1 can repress the activation of the *AtTRY* promoter by AtGL3 and AtTTG1. AtTTG1 inhibited the activation of the promoter of *AtCPC* by AtGL3 and AtGL1 (Pesch *et al.*, 2015). Consequently, CsMYB6 acts as a negative regulator of cucumber spine initiation, and attenuates the promoter activity of *CsTRY*, while some other proteins that interact with CsMYB6 may regulate the inhibition of the *CsTRY* promoter. Protein–protein interaction assays suggested that CsTRY also interacts directly with CsMYB6 protein, indicating that CsTRY may participate in the regulation of the inhibition of its promoter by CsMYB6. Recently, our research demonstrated that *CsTTG1* regulated fruit spine formation in cucumber (Chen *et al.*, 2016), which suggested that *CsTTG1* may also participate in the modulation of the activity of the *CsMYB6-CsTRY* complex. In conclusion, our results uncover a new role for the *CsMYB6-CsTRY* complex in regulating trichome initiation. However, the molecular mechanisms underlying how and why CsMYB6 represses *CsTRY* transcriptional activity require further study.

CsTTG1 regulated further differentiation of spines via interaction with CsGL1, while CsTTG1 seemed to be independent of Mict/CsGL1 in regulating fruit trichome initiation (Chen *et al.*, 2016). Overexpressing *CsMYB6* in the *csgl1* mutant caused a lower density of fruit trichomes; however, the morphology of the trichomes was not rescued, indicating that *CsMYB6* is involved only in the initiation of fruit trichomes (Fig. 4I–N). However, the expression of *CsMYB6* is strongly decreased in the *csgl1* mutant. The interaction between CsGL1 and CsTTG1 influences the expression of CsTTG1, which may affect the expression of CsMYB6. Future studies to obtain knockout transgenic lines of CsMYB6 and CsTTG1 by using CRISPR/Cas9 technology would help research into the molecular mechanism of trichome formation in cucumber.

## Supplementary data

Supplementary data are available at *JXB* online.

Fig. S1. Relative transcript abundance of *CsMYB6* in different tissues of wild-type and *csgl1* mutant.

Fig. S2. Morphological characterization of *CsMYB6*-overexpressing cucumber plants.

Fig. S3. Phenotypic analysis of *CsMYB6-RNAi* cucumber plants.

Fig. S4. Ectopic expression of *CsMYB6* in wild-type Arabidopsis caused the emergence of trichomes with two branches.

Table S1. Primers used in this study.

Table S2. Proteins interacting with CsMYB6.

Table S3. Effect of *CsMYB6* on trichome and its branch numbers on leaves.

Supplementary Figures and Tables
