# Supplementary material for: A CsMYB6-CsTRY module regulates fruit trichome initiation in cucumber
Source: J Exp Bot. 2018 Feb 8;69(8):1887–902. doi: 10.1093/jxb/ery047 (PMC6019040; doi:10.1093/jxb/ery047)
Supplement: Supplementary Figures and Tables [file ery047_suppl_supplementary_figures_and_tables.pdf]

Supplementary data

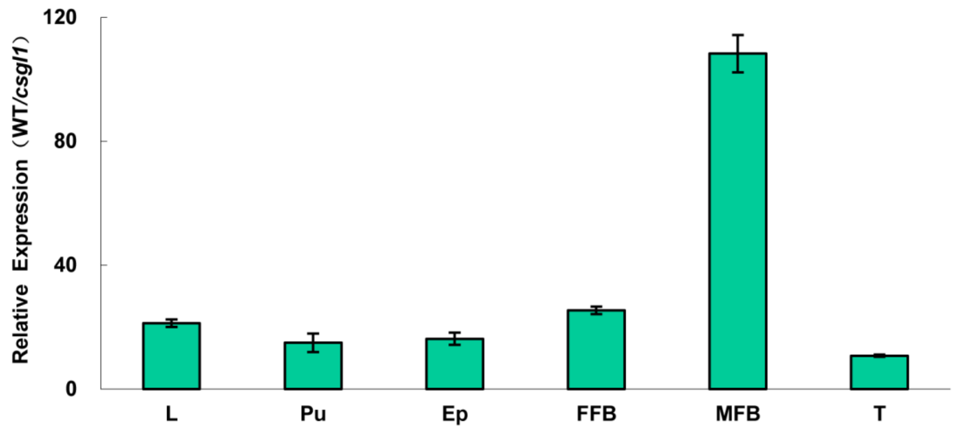

Fig. S1. Relative transcript abundance of *CsMYB6* in different tissues of WT and *csgl1* mutant. L, leaf; Pu, pulp; Ep, epidermis including fine spines; FFB, female flower bud; MFB, male flower bud; T, tendril.

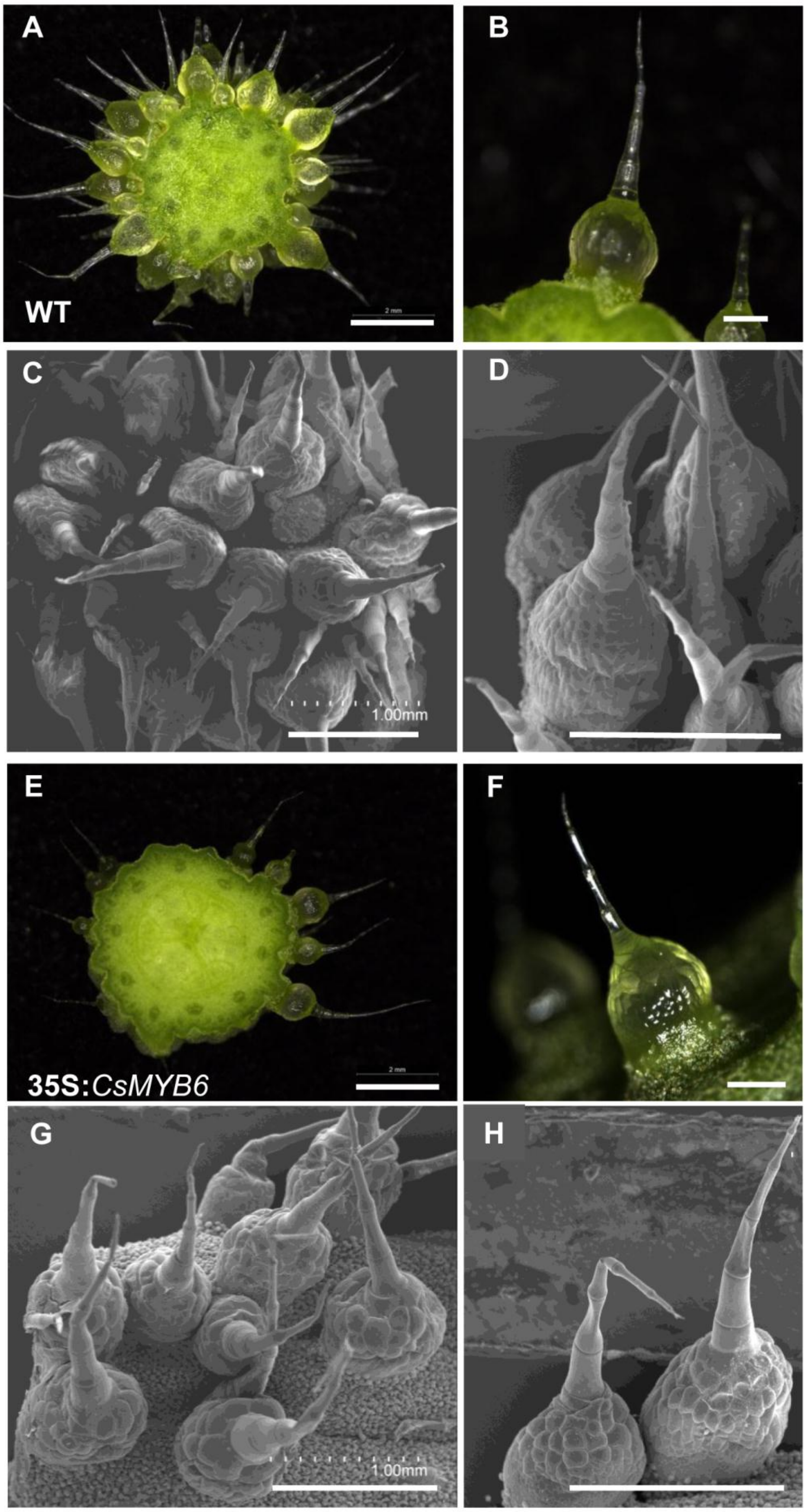

Fig. S2. Morphological characterization of *CsMYB6* overexpression cucumber plants. Morphology of young fruit of WT (A,C) and *CsMYB6* overexpression cucumber plants (E, G). Morphology of fruit spines in WT (B,D) and *CsMYB6*

overexpression cucumber plants (F, H). Bars, 2 mm (A,E); 500 μm (B,F); 1 mm (C,D,G,H).

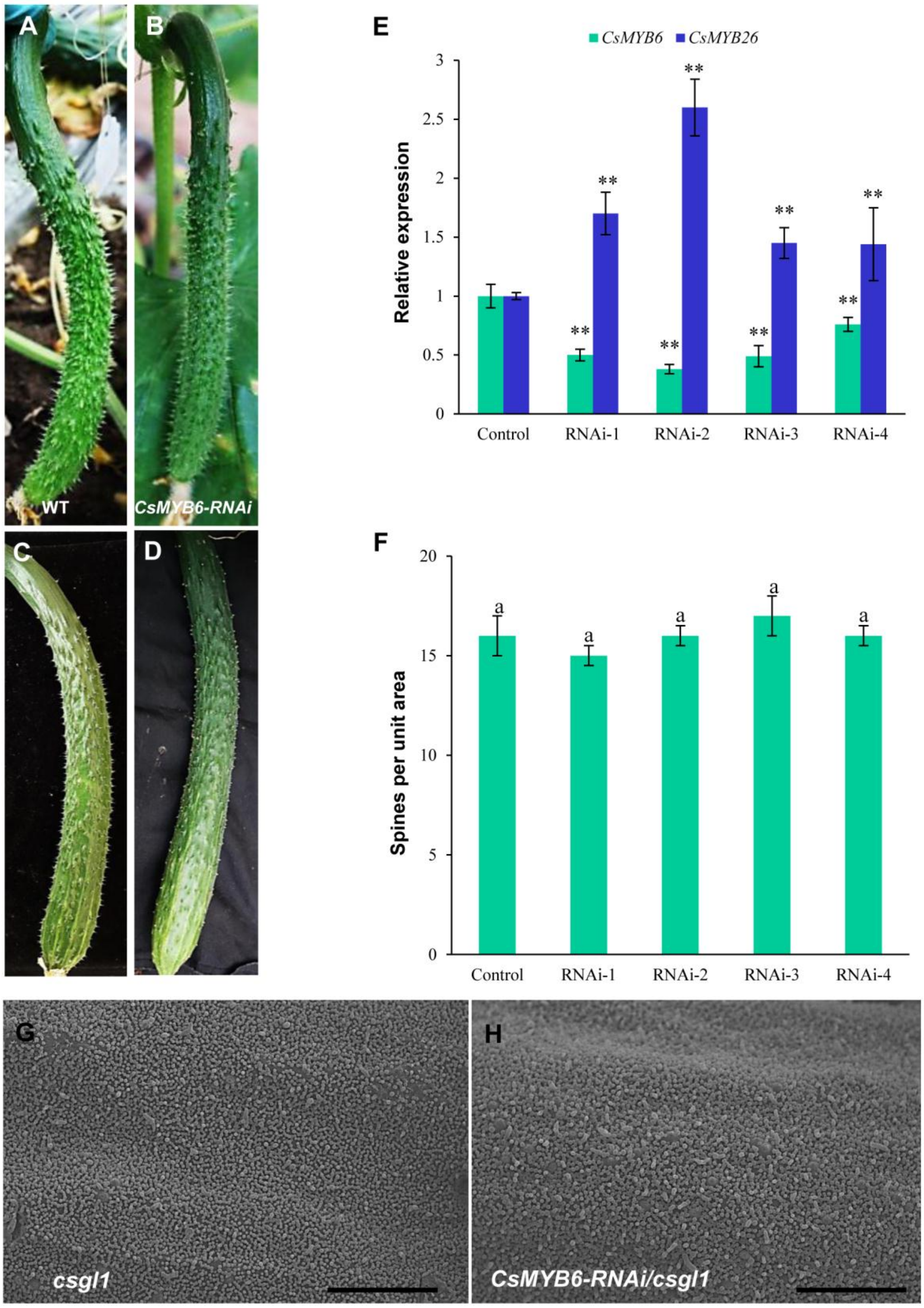

Fig. S3. Phenotypic analysis of *CsMYB6-RNAi* cucumber plants. Morphology of the fruits of WT (A,C) and *CsMYB6-RNAi* cucumber line (B,D). (E) qRT-PCR analyses of *CsMYB6* and *CsMYB26* in wild-type (WT) plants and RNAi transgenic plants. The cucumber *α-TUBULIN* (*TUA*) was used as an internal control, Error bars revealed the standard deviations of three independent replicates. (F) The number of trichome in wild-type and RNAi lines. Error bars represent +SE. Significant

differences were determined according to Duncan’s multiple range test ($P < 0.05$) or Student’s *t*-test (**P<0.01). Scanning electron microscopy images of the fruit surface from *csgl1* (G) and *CsMYB6-RNAi* /*csgl1* transgenic plants (H). Bars, 500 μm (G,H).

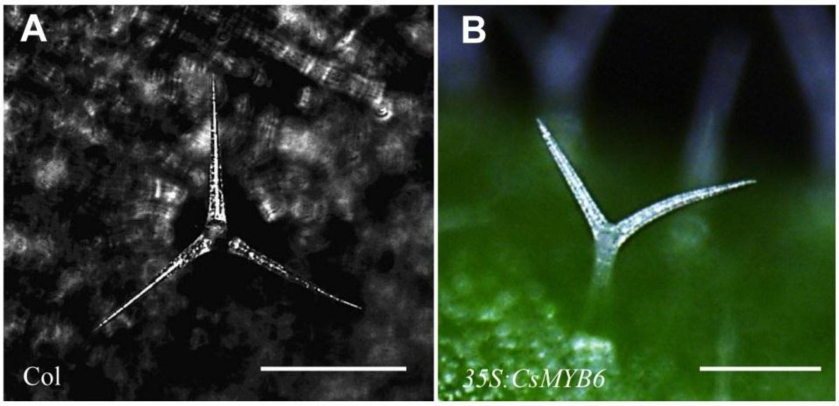

Fig. S4. Ectopic expression of *CsMYB6* in wild-type Arabidopsis caused the emergence of trichomes with two branches. Bars, 1 mm (A,B).

Table S1. Primer information used in this study.

**Primers for cloning**
CsMYB6-F 5'- ATGGGAAGGTCTCCTTACTGCG -3'
CsMYB6-R 5'- TCAGAATCTCAGGAATTCACCAAGA -3'
CsTRY-F 5'- ATGGACAATCATCGTCACCA -3'
CsTRY-R 5'- TCATCCTCTTCTTCTTTTTCCA -3'
**Primers for qRT-PCR**
q-CsMYB6-F 5'- CCAAAGCCGGTCTTGAGAGA -3'
q-CsMYB6-R 5'- GCTATGGCAGACCACCTGTT -3'
CsTRY-F 5'- ATGGATAACACTGACCGTCG -3'
CsTRY-R 5'- CTAGGAAGGATAGATAG -3
Mict -F 5'- AACCGTCGTGCAAGGTGGA -3'
Mict -R 5'- TCTGGTGGCGTCGTGTAGTG -3'
CsTTG1-F 5'- ATGGAACACGCAGCCTCCAG -3'
CsTTG1-R 5'- TCAAACTTTCAAAAGCTGCATTTTG-3
TUA-F 5'-ACGCTGTTGGTGGTGGTAC-3'
TUA-R 5'-GAGAGGGGTAAACAGTGAATC-3'
**Primers for *in situ* probes**
CsMYB6-SP6
5'-GATTTAGGTGACACTATAGAATGCTGGAAAGTTCAGTTTGCAGGAAG -3'
CsMYB6-T7
5'- TGTAATACGACTCACTATAGGGATCATCGGCAACACCCCTCT -3'
CsTRY-F
5'-GATTTAGGTGACACTATAGAATGCTACAATCATCGTCACCAGAAACC -3'
CsTRY-R
5'- TGTAATACGACTCACTATAGGGATCCCACCTATCTCCAATCAGC -3
**Primers for GUS construct**

ProCsMYB6-F 5'- AACTGCAGTGTGGACAGAAAATAATGTAGTAAA -3'

ProCsMYB6-R 5'- CGGGATCCTTGGATTTATCACAATTTTTGTC -3'

CsTRY-F

5'-GATTTAGGTGACACTATAGAATGCTACAATCATCGTCACCAGAAACC -3'

CsTRY-R

5'- TGTAATACGACTCACTATAGGGATCCCACCTATCTCCAATCAGC -3

Primers for GFP construct

G-CsMYB6-F 5'- GCTCTAGAATGGGAAGGTCTCCTTACTGCG -3'

G-CsMYB6-R 5'- TCCCCCGGGGAATCTCAGGAATTCACCAAGA -3'

CsTRY-F 5'- GCTCTAGAATGGACAATCATCGTCACCAGAAA-3'

CsTRY-R 5'- TCCCCCGGGTCCTCTTCTTCTTTTTCCAAACCCT -3

Primers for *CsMYB6* overexpression construct

O-CsMYB6 5'- GCTCTAGAATGGGAAGGTCTCCTTACTGCG -3'

O-CsMYB6 5'- TCCCCCGGGTCAGAATCTCAGGAATTCACCAAGA -3'

CsTRY-F 5'- GCTCTAGAATGGACAATCATCGTCACCA -3'

CsTRY-R 5'- TCCCCCGGGTCATCCTCTTCTTCTTTTTCCA -3

Primers for *CsMYB6-RNAi* construct

I-CsMYB6-F1 5'- AGGCGCGCCCACTCACAAACCAACGAAGAAC -3'

I-CsMYB6-R1 5'- ATTTAAATATCATCGGCAACACCCCTCT -3'

I-CsMYB6-F2 5'- GACTAGTCACTCACAAACCAACGAAGAAC -3'

I-CsMYB6-R2 5'- CGGGATCCATCATCGGCAACACCCCTCT -3'

Primers for PCR identification

O-CsMYB6-Fi 5'- CTATCCTTCGCAAGACCCTTC -3'

O-CsMYB6-Ri 5'- GCTATGGCAGACCACCTGTT -3'

CsTRY-F 5'- CTATCCTTCGCAAGACCCTTC -3'

CsTRY-R 5'- CTAGGAAGGATAGATAG -3

Primers for yeast two-hybrid construct

MPA1-F 5’-GGAATTCCATATGATGGGAAGGTCTCCTTACTGC-3’

MPA1-R 5’-CGCGGATCCATTTCTGATCTCCTAGTTGTCGAAA-3’

MPA2-F 5’-GGAATTCCATATGATGGGAAGGTCTCCTTACTG-3’

MPA2-R 5’-CGCGGATCCGACTAATGATCTTAAAGTTAAATTTTC-3’

MPA3-F 5’-GGAATTCCATATGATGGGAAGGTCTCCTTACTGCG-3’

MPA3-R 5’-CGCGGATCCACTATCAAACATATATGATTTTTGTTTA-3’

MPA4-F 5’-GGAATTCCATATGGTACGTCTACGTTTTGAATTTTCG-3’

MPA4-R 5’-CGCGGATCCTGTCATCGACTAATGATCTTAAAGT-3’

MPA5-F 5’-GGAATTCCATATGGAAAAACATATAGGTCTTGAGAGATG-3’

MPA5-R 5’-CGCGGATCCTGACACAACCTGAAGAAGAAGGA-3’

MPA6-F 5’-GGAATTCCATATGAAACATATAGGTCTTGAGAGATGTGGG-3’

MPA6-R 5’-CGCGGATCCGAATCTCAGGAATTCACCAAGA-3’

MPA7-F 5’-GGAATTCCATATGCGGTGTTTTAGGTGGTCTGCCATAG-3’

MPA7-R 5’-CGCGGATCCGAATCTCAGGAATTCACCAAGA-3’

MPA8-F 5’-GGGAATTCCATATGCGGTGTTTTAGGTGGTCTGCCATAG-3’

MPA8-R 5’-CGCGGATCCTGACACAACCTGAAGAAGAAGGAATTG-3’

MPA9-F

5’-GGGAATTCCATATGCGCGGATCCATGAAGGCGGTGTGGAGTCC-3’

MPA9-R 5’-CGCGGATCCGAATCTCAGGAATTCACCAAGAATTG-3’

TRY-BK-F 5’-TGATCTCAGAGGAGGACCTGCATATGATGGACAATCATCGTC

ACCAGAA-3’

TRY-BK-R 5’-TGCAGGTCGACGGATCCCCGGGAATTCTCCTCTTCTTCTTTTT

CCAAAC-3’

TRY-AD-F 5’-CCATACGACGTACCAGATTACGCTCATATGATGGACAATCATC

GTCACCA-3’

TRY-AD-R 5’-GATCCCGTATCGATGCCCACCCGGGTGGAATTCTCCTCTTCTT

CTTTTTCCA-3’

Primers for BiFC construct

B-IND-F 5'-TGCTCTAGA ATGGAAAATGGTATGTATAAAAAG -3'

B-IND-R 5'-CGCGGATCC GGGTTGGGAGTTGTGGTA -3'

B-SPT-F 5'-TGCTCTAGA ATGATATCACAGAGAGAAGAAAGA -3

B-SPT-R 5'-CGCGGATCC AGTAATTCGATCTTTTAGGTCAG -3'

B-CsMYB6-F 5'-CGGGATCCATGGGAAGGTCTCCTTACTGCG-3'

B-CsMYB6-R 5'-TCCCCCGGGGAATCTCAGGAATTCACCAAGA3'

B-TRY-F 5'CGGGATCC ATGGACAATCATCGTCACCA3'

B-TRY-R 5'-TCCCCCGGGGTCCTCTTCTTCTTTTTCCA3'

Primers for yeast one-hybrid construct

MBS1-pAbAi – F

5'-AAGCTTGAATTCGAGCTCTACAAGTCTACCAACCCAACAAGTC-3'

MBS1-pAbAi - R

5'-CATGCCTCGAGGTCGACGTTTCTTCAAAGATTAGAGGCAGCA-3'

MBS2-pAbAi -*F*

5'-AAGCTTGAATTCGAGCTCAGTTGGTGGGTGCTGAGCTTAT-3'

MBS2-pAbAi -*R*

5'-CATGCCTCGAGGTCGACTTGTTGGGTTGGTAGACTTGTAGTT-3'

MBS3-pAbAi -*F* 5'- AAGCTTGAATTCGAGCTCGCATGTCCTTGCTTGGG-3'

MBS3-pAbAi -*R*

5'- CATGCCTCGAGGTCGACATCTCACCCATAAATATTATATTCAA-3'

Primers for EMSA assay

MYB6-PGE - F

5'- GGATCTGGTTCCGCGTGGATCCATGGGAAGGTCTCCTTACTGCG-3'

MYB6-PGE -A

5'- TCAGTCAGTCACGATGAATTCGAATCTCAGGAATTCACCAAGA-3'

Biotin1- F- 5'- biotin-CCAACCCAACAAGTCAACCAACAAACTTAC-3'

Biotin1-A -5' -biotin- GTAAGTTTGTTGGTTGACTTGTTGGGTTGG-3'

Biotin2- F- 5' -biotin- CCCTGTAAGTTGTCAACTACAAGTCTACCA-3'

Biotin2-A -5’ -biotin- TGGTAGACTTGTAGTTGACAACTTACAGGG-3’

Biotin3- F- 5’ -biotin- CTAATTAGTTGTCAACTCATGATCATTGAA-3’

Biotin3-A -5’ -biotin- TTCAATGATCATGAGTTGACAACTAATTAG-3’

Cold-P1- F-5’- CCAACCCAACAAGTCAACCAACAAACTTAC -3’

Cold-P1- A-5’- GTAAGTTTGTTGGTTGACTTGTTGGGTTGG -3’

Cold-P2- F-5’- CCCTGTAAGTTGTCAACTACAAGTCTACCA -3’

Cold-P2- A-5’- CCCTGTAAGTTGTCAACTACAAGTCTACCA -3’

Cold-P3- F-5’- CTAATTAGTTGTCAACTCATGATCATTGAA -3’

Cold-P3- A-5’- TTCAATGATCATGAGTTGACAACTAATTAG -3’

Primers for Dual-LUC assay

TRYp1-LUC-F-5’-TTGATATCGAATTCCTGCAGCCCGGGCTAAGGGACGAGTAGGTTAA-3’

TRYp1-LUC-A-5’-GCGGTGGCGGCCGCTCTAGAACTAGTAAGATTAGAGGCAGCACAA-3’

MYB6-62SK-F-5’-GCGGTGGCGGCCGCTCTAGAACTAGTATGGGAAGGTCTCCTTACTGCG-3’

MYB6-62SK-A-5’-TTGATATCGAATTCCTGCAGCCCGGGTCAGAATCTCAGGAATTCACCAAGA-3’

Primers for COIP assay

MYB6-His-F-5’-AGCTTCTGCAGGGGCCCGGGATGGGAAGGTCTCCTTACTGCG-3’

MYB6-His-A-5’-ATGGTGATGGTGATGATGGAATCTCAGGAATTCACCAAGA-3’

TRY-HA-F-5’-AGCTTCTGCAGGGGCCCGGGATGGACAATCATCGTCACCAGAA-3’

TRY-HA-F-5’-AGCGTAGTCTGGGACGTCGTATGGGTATCCTCTTCTTCTTTTTCCAAAC-3’

Table S2. The proteins interacted with CsMYB6.

Gene ID	GO
**Csa5M139610.1**	**CsTRY**
Csa3M824850.1	CsMYB6
Csa5M583310.1	ubiquitin-associated (UBA)/TS-N domain-containing protein
Csa5M604380.1	ATUBC2 (UBIQUITING-CONJUGATING ENZYME 2); ubiquitin-protein ligase
Csa1M084320.1	histone H4
Csa7M073750.1	cyclin binding / cyclin-dependent protein kinase inhibitor
Csa3M238100.2	oligopeptide transporter OPT family,Probable metal-nicotianamine transporter
Csa5M266830.1	protein synthesis initiation factor eIF2 gamma The mRNA is cell-to-cell mobile.
Csa6M106180.1	nuclear RNA-binding protein (RGGA)
Csa1M050240.1	glyceraldehyde-3-phosphate dehydrogenase
Csa3M829270.1	pathogen-responsive alpha-dioxygenase, putative
Csa3M816160.1	alpha-N-arabinofuranosidase/ hydrolase, acting on glycosyl bonds / xylan 1,4-beta-xylosidase
Csa3M764560.1	Carboxy-lyase, putative,Putative lysine decarbxylase
Csa6M518060.5	drought-responsive family protein
Csa3M710870.1	WRKY40; transcription factor
Csa6M526230.1	(WRKY DNA-binding protein 35); transcription factor
Csa1M043100.1	zinc finger (C3HC4-type RING finger) family protein
Csa1M045550.1	Homeobox-leucine zipper protein, DNA binding / protein homodimerization/ sequence-specific DNA binding / transcription activator/ transcription factor

Table S3. Effect of *CsMYB6* on trichome and its branch numbers on leaves

Line	Percentage of trichome with different branchs(%) [a]					Trichome number per area [b]
	2	3	4	5	6	
nok	-	9.8	49.5	37	3.7	22 ±2
35S:CsMYB6::nok #1	-	10.1	62.5	27.4	-	16 ±4
35S:CsMYB6::nok #2	-	8.8	69	22.2	-	15 ±3
35S:CsMYB6::nok #3	-	6	70	24	-	16 ±2
35S:CsMYB6::nok #4	-	8.7	73.8	17.5	-	13 ±2
Col		89.75	10.25	-	-	21 ±2
35S:CsMYB6::Col #1	5.5	94.5	-	-	-	12 ±3
35S:CsMYB6::Col #2	7.5	90.6	1.9	-	-	9 ±3
35S:CsMYB6::Col #3	3.4	93.2	3.4	-	-	10 ±2
35S:CsMYB6::Col #4	6.6	92.1	1.3	-	-	13 ±2

a Percentage of trichomes. **b** The density of trichome .Trichomes were counted on leaves of the first leaf pair of five plants.
